# Limitations of linear load-velocity modeling for bench press performance in youth elite athletes

**DOI:** 10.3389/fspor.2026.1893029

**Published:** 2026-07-23

**Authors:** Sebastian Puschkasch-Möck, Martin Hillebrecht, Michael Keiner, Carl-Maximilian Wagner, Andreas Konrad, Konstantin Warneke

**Affiliations:** 1 Department of Exercise Science, Olympic Training and Testing Center of Hessen, Frankfurt, Germany; 2 University Sports Center, Carl von Ossietzky University Oldenburg, Oldenburg, Germany; 3 Department for Sport and Exercise Science, German University of Health and Sport, Ismaning, Germany; 4 Department for Exercise, Sport and Health, Leuphana University Lüneburg, Lüneburg, Germany; 5 Institute of Human Movement Science, Sport and Health, Karl-Franzens University Graz, Graz, Austria; 6 Institute of Sustainability Psychology, Leuphana University Lüneburg, Lüneburg, Germany

**Keywords:** load-velocity profiling, neural networks, one-repetition maximum, strength assessment, systematic bias

## Abstract

**Introduction:**

Load-velocity profiling is widely used to characterize strength and estimate maximal load in resistance exercises. Most applications rely on linear regression models, assuming a linear relationship between load and velocity. This assumption has rarely been examined in youth elite athletes, who show pronounced inter-individual variability in neuromuscular coordination and technique. The primary aim was to examine whether linear load-velocity models describe bench press performance in youth elite athletes and whether a neural network approach better captures individual load-velocity characteristics.

**Methods:**

Fifty-three youth elite athletes completed one-repetition maximum testing and a standardized load-velocity protocol. Linear regression models were compared with neural network models for systematic bias, agreement with measured one-repetition maximum, and estimation error.

**Results:**

Linear models underestimated maximal strength and showed limited agreement with measured values, indicating structural limits in representing load-velocity behavior. Neural network models reduced bias and estimation error and indicated population-specific nonlinearity of the load-velocity relationship. The best-performing neural network showed high agreement with measured values with low absolute and relative errors.

**Discussion:**

These findings indicate that linear load-velocity assumptions may be insufficient for strength assessment in youth elite athletes and highlight the relevance of nonlinear behavior in this population.

## Introduction

Resistance training plays a central role in both athletic performance development and health-oriented exercise prescription (1). In practice, a large proportion of programming decisions—such as load prescription, volume management, and fatigue monitoring—depends on an accurate determination of an individual's one-repetition maximum (1RM) (2). Despite its widespread acceptance as the criterion measure of dynamic strength, direct 1RM testing is time-consuming, requires extensive supervision, and may impose fatigue or injury risk, particularly in team-based or youth athletic settings (3, 4). This concern is especially relevant in adolescent populations, where ongoing neuromuscular development and variable maximal strength expression—combined with the practical realities of school or team-based training environments—create a particular need for efficient assessment methods. Although direct 1RM testing has been shown to be feasible and safe in youth populations (5), practical constraints in team-based settings and the additional value of understanding load-velocity relationships make indirect estimation methods particularly valuable in adolescent training contexts. Accordingly, substantial effort has been directed toward the development of indirect estimation methods that can provide valid 1RM predictions while minimizing the burden associated with maximal testing.

One prominent approach in this regard is load-velocity profiling, which rests on the assumption of a systematic relationship between external load and barbell movement velocity during resistance exercise (6, 7). Based on established force-velocity and power-load relationships of skeletal muscle, it is typically assumed that increasing external load is accompanied by decreasing movement velocity, enabling extrapolation of maximal strength from submaximal velocities. Linear regression models derived from a small number of lifted loads are therefore frequently used to estimate 1RM without requiring an actual maximal lift (4, 8, 9). Although this approach is conceptually appealing and pragmatically attractive, reported validity indices are inconsistent, and prediction errors of practically relevant magnitude have been documented in several studies (6, 7). This illustrates that this assumption may oversimplify the shape of the load-velocity relationship, which could vary across individuals and potentially across different loading regions of the curve.

A key limitation in the current literature lies not only in the magnitude of estimation error but also in the way it is evaluated. Many studies rely primarily on correlation-based indices to assess the relationship between predicted and measured 1RM (7, 10), despite well-established concerns that correlation does not quantify agreement or practical prediction accuracy (11, 12). In addition, validation protocols are often conducted under conditions in which the actual 1RM is already known, and prediction models are tested within the same population from which they were derived. This limits ecological validity, as in practical settings the true 1RM is unknown and predictions must generalize to individuals not included in model development (13). Beyond these methodological considerations, little attention has been given to how deviations from linearity are distributed across the load spectrum and whether these deviations follow systematic patterns.

Beyond methodological concerns, there are also theoretical reasons to question the adequacy of simple linear models. The form of the load-velocity or force-velocity relationship is influenced by neuromuscular activation strategies, movement technique, anthropometric characteristics, training status, and properties of the measurement device, all of which introduce interindividual variability and potential nonlinearities (14, 15). Assuming a single, generalizable linear function across athletes may therefore neglect population- or individual-specific curve characteristics and systematically bias 1RM prediction. These considerations motivate the exploration of more flexible modeling approaches capable of capturing complex multivariate relationships in load-velocity data (16–18).

Machine learning–based methods, and neural networks in particular, offer such flexibility by enabling nonlinear mapping between input variables and performance outcomes (13, 17). Nevertheless, to our knowledge, Balsalobre-Fernández and Kipp (17) have presented the only study to date applying neural network approaches to velocity-based 1RM prediction, and evidence regarding their capacity to meaningfully reduce both systematic and random errors remains limited.

Against this background, the present study aimed to evaluate whether neural network–based approaches can improve the fitting of bench press 1RM derived from load-velocity data in youth elite athletes compared with commonly used linear regression models. Particular emphasis was placed on the magnitude of both systematic and random error and on the practical relevance of the achieved accuracy for strength-training prescription. Specifically, the study examined whether neural network models are capable of reducing systematic bias in 1RM fitting relative to conventional linear regression approaches and whether they can decrease random error—expressed through indices such as mean absolute error, mean absolute percentage error, and limits of agreement—to a level that is practically meaningful in applied settings. Furthermore, we explored whether the inclusion of additional anthropometric and mechanically derived variables enhances fitting accuracy compared with models relying solely on barbell velocity data.

Based on the theoretical limitations of linear modeling and the greater flexibility of nonlinear multivariate approaches, it was hypothesized that neural network–based models would yield lower random error, smaller systematic bias, and narrower limits of agreement with directly measured 1RM than linear regression models. In addition, it was expected that neural networks incorporating additional anthropometric or derived mechanical input variables would outperform models relying exclusively on barbell velocity information.

## Materials and methods

### Design

This study employed a cross-sectional design to investigate the limitations of linear load-velocity modeling in explaining bench press performance in youth elite athletes. Fifty-three participants completed a standardized warm-up followed by a direct one-repetition maximum (1RM) assessment. To characterize individual load-velocity behavior, athletes performed submaximal bench press lifts at predefined percentages of their measured 1RM, following a standardized protocol to ensure consistent execution and velocity measurement. Linear regression models were applied to the load-velocity data to estimate 1RM, and fittings were compared with neural network models designed to capture potential nonlinearities in the load-velocity relationship and account for inter-individual differences in neuromuscular coordination and technique. Velocity data were collected using a linear position transducer, and fitting performance was evaluated using agreement statistics and error metrics.

### Participants

An *a priori* power analysis using G*Power, assuming a high correlation of *r* = 0.70 with *α* = 0.05 and *β* − 1 = 0.95, indicated a minimum required sample size of 40 participants. To further increase statistical power and the stability of estimation models, 53 youth elite athletes were recruited (27 male and 26 female; age 17.75 ± 1.46 years; height 1.75 ± 0.11 m; body mass 64.43 ± 9.15 kg). All athletes competed in biathlon or cross-country skiing at the highest national level for their age group and had prior experience with resistance training, including regular bench press exercises performed approximately one to two times per week. Training experience ranged from six months to four years.

All participants were free from musculoskeletal injury at the time of testing and refrained from strenuous training on the day of assessment. Written informed consent was obtained from each participant, and parental consent was additionally obtained for minors. The study was conducted in accordance with the Declaration of Helsinki and approved by the Ethics Committee of the German University of Health and Sport (DHGS-EK-2023-004).

### Testing procedure

Before testing, participants completed a standardized 10-min warm-up consisting of ergometer cycling at 60 revolutions per min followed by three sets of six to eight submaximal repetitions of the bench press. Following the warm-up, the 1RM test was performed. After a minimum rest period of 5 min, three submaximal loads corresponding to 30%, 50%, and 70% of the measured 1RM were lifted. For each load, two repetitions were performed, and participants were instructed to complete the concentric phase with maximal voluntary acceleration. The repetition with the highest mean concentric velocity was retained for further analysis. Participants were familiarized with all procedures beforehand to minimize learning effects and were instructed to avoid intensive exercise for 24 h prior to testing.

### Maximal strength determination via 1RM

Dynamic maximal strength of the bench press was determined using a standardized 1RM testing protocol. Participants began lifting at approximately 90%–95% of their estimated 1RM, and the external load was progressively increased in increments of 2%–5% until the load could no longer be successfully lifted. After two consecutive unsuccessful attempts, the heaviest successfully lifted load was recorded as the 1RM. Rest intervals of at least 5 min were provided between attempts to minimize fatigue, and no more than five maximal attempts were permitted per participant.

A trial was considered valid when several criteria were fulfilled. The participant was required to maintain a five-point body contact position, consisting of head, upper back, and buttocks in contact with the bench and both feet flat on the floor. At the end of the eccentric phase, the barbell was required to touch the chest with a brief pause before the concentric movement was initiated. The lift was completed when full elbow extension was achieved. Bouncing or uncontrolled use of the chest for elastic recoil was not permitted, and controlled chest contact consistent with standard powerlifting practice was required.

### Force-velocity profile with submaximal loads

Force-velocity profiling was conducted using submaximal loads corresponding to 30%, 50%, and 70% of the directly measured 1RM, in line with previous recommendations on the assessment of power output and load–velocity characteristics in resistance exercise (19, 20). After a controlled eccentric phase with brief chest contact, participants were instructed to perform the concentric phase as rapidly as possible with maximal intent. Two repetitions were executed at each load, separated by 2 min of rest, and the repetition displaying the highest velocity value was used for subsequent analyses.

Barbell velocity was measured with a TENDO Unit linear position transducer (TENDO Sports Machines; Trencin, Slovak Republic) attached to the barbell. This system has previously been presented to demonstrate acceptable validity and reliability for quantifying barbell movement velocity during resistance exercise (21). Customized software was used to compute velocity and power output across the full range of motion during each repetition.

### Linear regression models

Linear regression models were constructed on the basis of the widely used assumption of an inverse linear relationship between external load and mean concentric barbell velocity during resistance exercise (6, 7). Velocity data obtained at 30%, 50%, and 70% of 1RM served as input to individual regression models from which 1RM was estimated. Two common approaches to regression-based 1RM estimation were implemented. First, the LD0 model extrapolated the regression line to zero velocity, representing the theoretical point of maximal force production. Second, a minimal velocity threshold model estimated 1RM at predefined minimal concentric velocities ranging from 0.10 to 0.20 m·s^−1^, corresponding to velocities typically observed during near-maximal lifts (4, 17). These approaches were selected to enable direct comparison with previously published work in the field.

### Improving the 1RM fitting using a neural net approach

To address potential limitations of linear regression modeling, artificial neural networks were used to capture nonlinear relationships within the load-velocity data. Neural networks were constructed and trained using MemBrain, an open-source neural network simulator (https://membrain-nn.de/index.htm) that has been employed in previous scientific applications (22). Several neural network architectures were examined to determine whether increasing model complexity improves fitting accuracy.

Models varied with respect to the type and number of input variables. Configurations included networks trained exclusively with barbell velocity values, networks integrating both velocity and lifted load information, and networks incorporating derived variables such as the load-velocity quotient. Additional models included anthropometric variables, namely body mass and body mass index, to evaluate whether the integration of subject characteristics enhanced fitting precision. Across configurations, networks comprised between two and five input neurons and between nine and eleven hidden neurons, with a single output neuron representing the estimated 1RM. Networks were trained using a standard backpropagation algorithm with randomly initialized weights. Each network configuration was trained ten times, and the model demonstrating the lowest error was retained for further analysis. Learning rate, activation functions, stopping criteria, and regularization strategies were selected with the aim of minimizing overfitting and improving generalizability to new data (13).

### Statistical analysis

All statistical analyses were performed using JAMOVI and the “semlmatrix,” “blandr,” and “regression” modules. The Shapiro–Wilk test was applied and verified normal distribution of the data. Descriptive statistics for all main variables are presented as mean ± standard deviation.

Agreement between the directly measured 1RM and the extrapolated 1RM values obtained from both linear regression models and neural network approaches was evaluated using several complementary indices. Pearson correlation coefficients were calculated to quantify linear associations, and intraclass correlation coefficients (single measures, two-way mixed effects, absolute agreement model) with 95% confidence intervals were determined to assess relative agreement. Since correlation does not by itself provide information on interchangeability, Bland–Altman analyses were conducted to examine systematic bias and limits of agreement between measured and extrapolated values (23, 24). Paired-samples t-tests were applied to test for statistically significant systematic differences.

To quantify fitting accuracy, both absolute and relative error indices were calculated. Mean absolute error (MAE) was used to express the average absolute difference between extrapolated and measured 1RM values (25), whereas mean absolute percentage error (MAPE) quantified the relative magnitude of fitting error with the measured 1RM as reference (26, 27). Additionally, model performance was evaluated for the best performing net using a stratified 5-fold cross-validation procedure. For each fold, MAE, MAPE, and ICC between predicted and observed 1RM values were calculated. For all analyses, the level of statistical significance was set at *p* < 0.05. No adjustment for multiple comparisons was applied, as the primary aim of the statistical analyses was the descriptive and comparative evaluation of model performance rather than confirmatory hypothesis testing. Accordingly, interpretation of results was primarily based on effect size and agreement metrics (MAE, MAPE, ICC, and Bland–Altman limits of agreement), while *p*-values were reported as supplementary inferential information.

## Results

**Figure 1:**
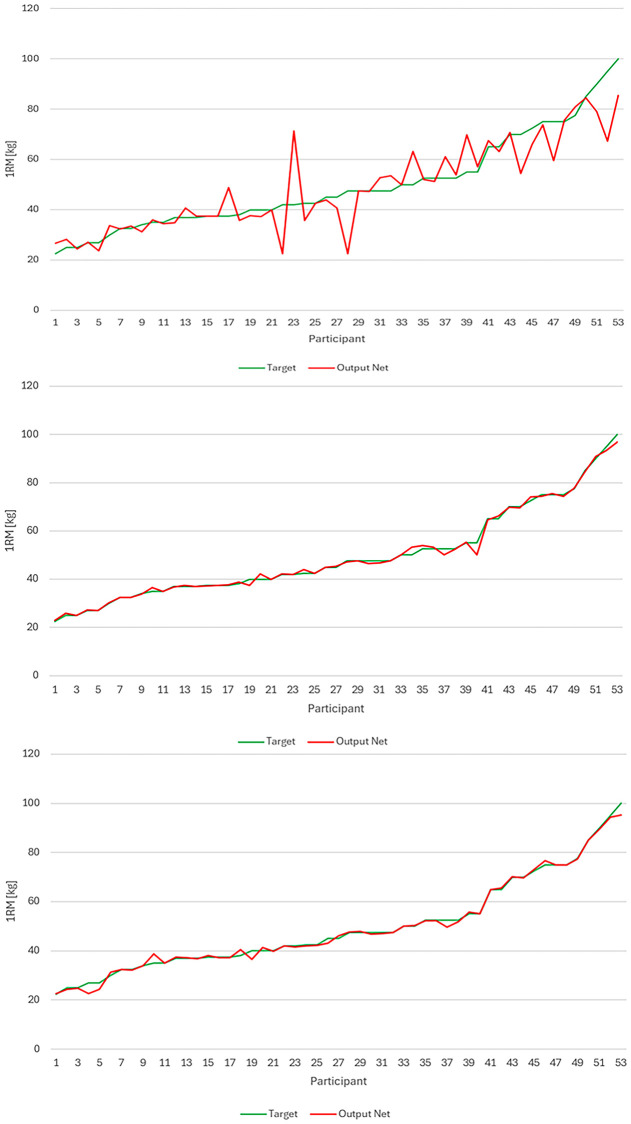
Illustration of the back propagation process to find a fitting model in 1RM prediction.

**Figure 2:**
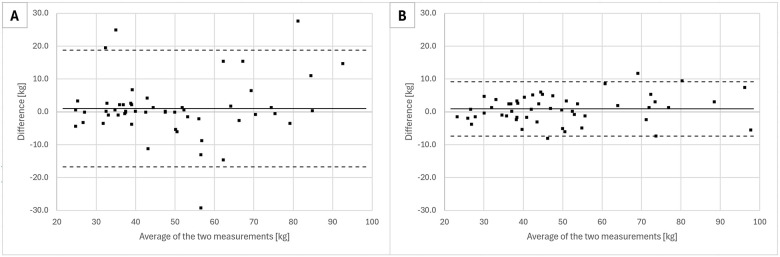
BA-Plots to illustrate the limits of agreement and individual scattering around the systematic bias line including detection of heteroscedasticity using the LD0 **(A)** and the best neural net approach 5 interneurons with the weight of 30%, 50% and 70% of the 1RM divided through the velocity (modified velocity values, body mass and BMI) as a suggested weight-velocity quotient **(B)** and the actually measured 1RM.

The mean measured 1RM across all participants amounted to 49.79 ± 18.60 kg. Extrapolated 1RM values obtained from the different linear regression models and neural network approaches, together with indices of agreement and error metrics, are summarized in Table 1. Bland–Altman plots illustrating individual levels of agreement are provided in Figures 1, 2.

**Table 1:** Descriptive and agreement statistics for the actually measured 1RM and the predicted 1RM, dependent on the chosen calculation model.

Calculation model	1RM	Predicted 1RM	Correlation coefficient (*p*-value)	ICC (95%CI)	Limits of Agreement	MAE in kg	MAPE in %	Systermatic bias (t; *p*-value); df = 52
1RM vs. LinRegLD0	49.79 ± 18.60	49.20 ± 18.13	0.93 (<0.001)	0.98 (0.96–0.99)	−7.37 to 8.54	3.18	6.54	0.16 (1.546; 0.87)
1RM vs. LinRegvMin0.1		46.31 ± 17.20	0.94 (<0.001)	0.96 (0.79–0.98)	−4.53 to 11.48	4.14	8.05	2.79 (6.439; 0.003)
1RM vs. LinRegvMin0.2		43.42 ± 16.27	0.94 (<0.001)	0.91 (0.15–0.98)	−2.14 to 14.87	6.32	12.40	5.74 (10.721; <0.001)
1RM vs. Neural net 3		48.78 ± 17.55	0.88 (<0.001)	0.88 (0.80–0.93)	−18.77 to 16.77	5.40	10.63	1.25 (1.037; 0.43)
1RM vs. Neural net2 reg.		49.72 ± 18.40	0.99 (<0.001)	0.99 (0.978–0.993)	−5.98 to 5.84	2.11	4.13	−0.07 (0.804; 0.41)
1RM vs Neural net 1RM/v		49.76 ± 18.68	0.999 (<0.001)	0.999 (0.998–0.999)	−1.52 to 1.57	0.56	1.43	0.02 (0.288; 0.813)

### Linear regressions to extrapolate the tested 1RM

Across all linear regression approaches, strong correlations were observed between measured and extrapolated 1RM values, with Pearson correlation coefficients ranging from 0.93 to 0.94 (*p* < 0.001). Intraclass correlation coefficients indicated high relative agreement (ICC = 0.91–0.98). However, despite these high correlation-based indices, notable estimation errors were present. Mean absolute error ranged from 3.18 to 6.32 kg, corresponding to mean absolute percentage errors between 6.54% and 12.40%, using the measured 1RM of 49.79 kg as reference.

Systematic bias differed across models. The minimal velocity threshold approach demonstrated a statistically significant underestimation of the measured 1RM, whereas the LD0 method did not differ significantly from the directly measured value. Nevertheless, even for the LD0 model, random error remained considerable, with a mean absolute error of approximately 3.2 kg (6.5%). Limits of agreement ranged from −7.4 to 8.5 kg, indicating substantial interindividual variability. These results indicate that, although linear models show high relative reliability, the magnitude of random and systematic error is sufficiently large to limit their practical applicability for precise load prescription.

### Neural net approach

Neural network–based fitting models demonstrated variable performance depending on model architecture and input parameters (Figure 1). The network trained exclusively with velocity data from the three submaximal loads was unable to meaningfully reduce fitting error compared with linear regression approaches. This model produced a systematic underestimation of 1.25 kg and a mean absolute error of 5.40 kg, corresponding to a mean absolute percentage error of 10.63%, which was even higher than that observed in the best-performing linear regression model.

In contrast, the network using two input neurons that integrated velocity information with lifted loads yielded markedly improved fitting accuracy. In this configuration, systematic estimation error was reduced to −0.07 kg and no statistically significant bias was detected. Random error was substantially smaller than in the linear regression models, with a mean absolute error of 2.11 kg and a mean absolute percentage error of 4.13%, based on a reference 1RM of 49.74 kg. Limits of agreement ranged from −5.98 to 5.84 kg. Both Pearson and intraclass correlation coefficients indicated excellent relative agreement (*r* = 0.998; ICC = 0.998).

The best performance was achieved by the neural network model that incorporated modified input variables, specifically the quotient of lifted mass and corresponding barbell velocity for the 30%, 50%, and 70% loads, supplemented by body mass and body mass index. In this configuration, the measured 1RM of 49.79 ± 18.60 kg was extrapolated with a mean estimated 1RM of 49.76 ± 18.68 kg. Relative agreement indices reached *r* = 0.999 and ICC = 0.999. Systematic bias was negligible (mean difference = 0.002 kg) and random error was minimal, with a mean absolute error of only 0.56 kg and a mean absolute percentage error of 1.43%. Limits of agreement were narrow, ranging from −1.52 to 1.57 kg. The corresponding Bland–Altman plot is shown in Figure 2 and illustrates substantially reduced dispersion around the bias line when compared with the LD0 linear regression model.

Overall, neural network approaches demonstrated the capacity to reduce both systematic and random error in 1RM fitting when appropriate input features were included, particularly when derived mechanical indices and anthropometric variables were provided. Models based solely on velocity information were insufficient to improve accuracy, whereas models integrating additional load-dependent and subject-specific characteristics yielded substantially enhanced agreement with the measured 1RM.

**Figure 3:**
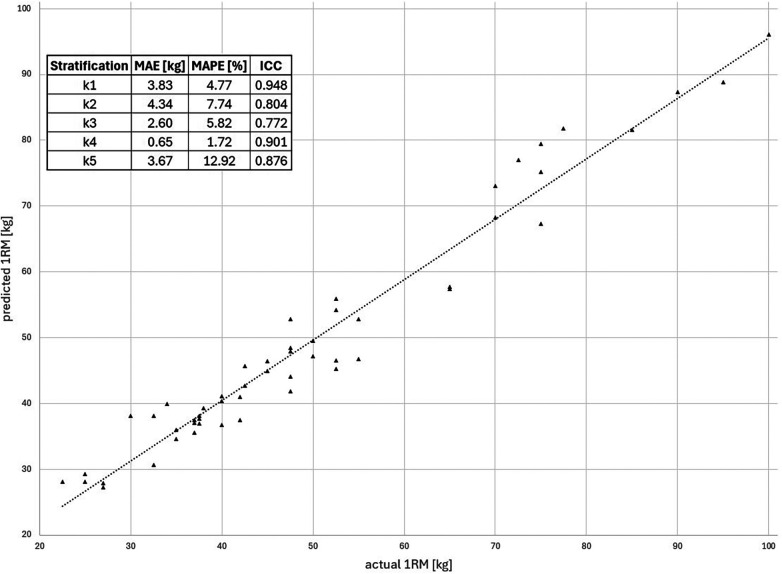
Summative scatter plot and statistical metrics of the stratified 5-fold cross-validation procedure.

The stratified 5-fold cross-validation demonstrated an overall good predictive performance of the artificial neural network (Figure 3). Across the five validation folds, the mean MAE was 3.02 kg, the mean MAPE was 6.59%, and the mean ICC was 0.860, with fold-specific values ranging from 0.65 to 4.34 kg, 5.82% to 12.92%, and 0.772 to 0.948, respectively.

## Discussion

This study is the first to apply neural network–based machine learning approaches with explicit consideration of both systematic and random error components to extrapolate bench press 1RM from submaximal load-velocity data. The principal finding was that linear regression approaches systematically underestimated 1RM in youth elite athletes, whereas neural network models demonstrated substantially improved agreement when additional load-dependent and anthropometric information was provided. The best-performing neural network configuration markedly reduced fitting error to a degree that appears practically relevant for strength diagnostics and load prescription.

Youth athletes are characterized by ongoing neuromuscular maturation, including incomplete optimization of motor unit recruitment and intermuscular coordination compared with adults (28). In parallel, growth-related changes in limb proportions and segmental lever arms may introduce additional biomechanical variability in force production (28). Even in elite youth athletes, high sport-specific skill levels may coexist with relatively less stable maximal strength expression, potentially reflecting an imbalance between technical proficiency and foundational strength capacity (29). Together, these factors may contribute to increased variability in force expression at higher loading intensities, where technical compensation strategies and velocity loss become more pronounced. This is consistent with the present findings, in which neural network models showed improved performance relative to linear approaches under conditions likely reflecting greater inter-individual variability.

In line with previous studies, the linear regression models examined in this work showed high correlation coefficients and intraclass correlation coefficients between measured and extrapolated 1RM values (4, 6, 7), yet still produced fitting errors of practically relevant magnitude. This finding supports earlier reports showing that linear models tend to underestimate true 1RM values and that considerable random error accompanies these estimates (11, 30). This finding supports earlier reports showing that linear models tend to underestimate true 1RM values and that considerable random error accompanies these estimates (11, 30). This was reflected in a consistent underestimation of true 1RM, particularly in the minimal velocity threshold model, alongside substantial random scatter in the LD0 approach. Notably, these limitations also indicate that barbell velocity alone may not fully capture biomechanical and neuromuscular factors determining maximal strength, such as inter-individual differences in technique, anthropometry, and force production strategy.

The practical consequences of such error magnitudes should not be underestimated. Underestimation of 6%–12% in 1RM prediction, as found in the linear models in this study, would directly influence prescribed relative loads in resistance training, thereby altering expected training stimuli and potentially increasing the risk of overuse or insufficient loading. This is particularly relevant for elite athletes, where fine-grained load adjustments are necessary, as well as in youth and rehabilitation settings, where safety and progressive overload are critical considerations (2, 11, 12, 31). Moreover, these errors imply that submaximal barbell velocity alone may not fully capture the mechanical and neuromuscular determinants of load tolerance and force production, such as individual leverages, or activation patterns.

Neural network models showed clear dependence on the type of input data. The network relying exclusively on barbell velocity values did not improve fitting accuracy and performed worse than the best linear model. This suggests that submaximal velocity alone does not sufficiently capture the multifactorial determinants of maximal strength capacity. When additional load information was included, fitting accuracy improved substantially and systematic bias was largely eliminated. These findings are consistent with previous suggestions regarding the suitability of machine learning approaches for modeling complex load-velocity relationships (17).

The most accurate fittings were obtained from the neural network model incorporating modified parameters, particularly the quotient of lifted mass and achieved velocity across submaximal loads, supplemented by body mass and body mass index. These variables likely provided indirect, scaling-related information reflecting inter-individual differences in body size and external load tolerance rather than direct biomechanical determinants of movement mechanics. In this sense, they may have assisted the model in capturing individual differences in performance expression across relative loading conditions. The nearly absent systematic bias, minimal mean absolute error, and extremely narrow limits of agreement observed in this model demonstrate the potential of neural networks to overcome limitations inherent in simple two-parameter linear regression approaches. In this context, differences between predicted and measured 1RM across models may tentatively indicate deviations from linearity primarily in the higher-load range. However, such effects were not explicitly localized, and the present analysis does not provide load-specific evidence for the lower-load domain.

Despite these promising results, several methodological considerations must be emphasized. The exceptionally high agreement indices (ICC ≈ 0.999) and extremely small error values inevitably raise concern about possible overfitting, especially given the limited sample size of 53 participants. Neural networks, though powerful, are sensitive to data volume and heterogeneity, and their performance may not generalize to unseen individuals without external validation. While the present study addressed this issue by applying a stratified 5-fold cross-validation, full resolution of overfitting concerns requires validation in independent populations and under conditions where true 1RM is not known *a priori* (13). Given the relatively small sample size, some variability in model performance across the validation folds was expected, and the findings should therefore be interpreted with appropriate caution.

A further methodological consideration concerns the definition of submaximal testing intensities as percentages of a previously measured 1RM. While this approach allows controlled comparison between prediction models, it does not fully reflect practical scenarios in which the true 1RM is unknown, and predictions must generalize to previously unseen individuals (13). In addition, the sample size of 53 participants limits the robustness of neural network training and the generalizability of the findings. Future studies should evaluate model performance in larger and more diverse cohorts and under conditions where the 1RM is truly unknown, to better approximate real-world application. More advanced machine learning architectures, such as deep learning networks, could further improve predictive accuracy, but their practical implementation requires careful consideration of computational complexity and data requirements. Moreover, the identification and inclusion of key input variables may enhance performance: in this study, the mass-velocity quotient, body mass, and body mass index yielded the lowest random and systematic errors. Incorporating additional mechanical or anthropometric factors, such as joint angles, lever arms, or individual neuromuscular coordination characteristics, could further reduce bias and error in 1RM prediction, thereby increasing the practical utility of velocity-based assessments.

Finally, the findings of the present study contribute to the ongoing discussion regarding the assumed linearity of the load-velocity relationship. Although linear models are intuitively appealing and easy to implement, the systematic underestimation and considerable random error identified here and elsewhere suggest that linear approximations may not adequately reflect underlying neuromuscular and biomechanical mechanisms (15, 30, 32). Individual differences in technique, anthropometry, neural activation strategies, and measurement device characteristics likely lead to nonlinear and subject-specific relationships that require more flexible modeling approaches. In particular, variations in leverages, muscle coordination, and barbell velocity profiles across submaximal loads may explain why linear models systematically misestimate maximal strength. Neural networks represent one such approach, but their adoption must be accompanied by rigorous validation and consideration of practical feasibility.

In summary, this study demonstrates that conventional linear regression approaches to velocity-based 1RM estimation exhibit meaningful systematic and random errors that limit their practical utility. Neural network–based models, when supplied with appropriate input variables, substantially reduced fitting error and improved agreement with directly measured 1RM values. These results position neural networks as a promising avenue for improving strength diagnostics and training load prescription. Nonetheless, given the small sample size and absence of external validation, the present findings should be regarded as preliminary. Further research incorporating larger datasets, alternative machine learning techniques, cross-validation procedures, and real predictive scenarios is required to determine whether neural networks can yield sufficiently robust and generalizable predictions for routine use in sports performance and rehabilitation settings.

While incorporating additional parameters may complicate practical implementation initially, it could significantly enhance prediction accuracy and reduce the risk of overestimating performance capacity. Importantly, our analysis was limited to the bench press, and movement-specific factors may further influence the predictions.

As with all empirical research, several limitations have to be considered when interpreting the results. One key limitation of the present study is the absence of evaluation in an independent dataset. All models were developed and evaluated within the same sample, which restricts conclusions regarding their generalizability to independent cohorts. Although agreement statistics indicated strong performance of the best neural network model, its stability and predictive accuracy in unseen populations remain to be confirmed. A second limitation relates to the use of relative loading intensities (30%, 50%, and 70% of the individually determined 1RM) to define the submaximal testing conditions. While this approach ensured standardized between-subject comparison, it introduces a degree of methodological dependency on the directly measured 1RM. Consequently, the present findings may not fully reflect applied scenarios in which true maximal strength is unknown and load prescription is based on estimated rather than measured values. Finally, it should be acknowledged that the sample size, while appropriate for the primary statistical analyses, is relatively small from a machine learning perspective and may limit the robustness of model training and parameter stability. In addition, multiple statistical tests were conducted across different models and agreement metrics. However, these analyses were primarily descriptive and aimed at quantifying predictive performance and agreement rather than testing confirmatory hypotheses. Therefore, no adjustment for multiple comparisons was applied, as the interpretation of results was based on effect size measures (e.g., MAE, MAPE, ICC, and limits of agreement) rather than statistical significance testing.

## Conclusions

While simplified mathematical models cannot fully replace direct 1RM testing, more sophisticated computational approaches and machine learning systems can be integrated with sensor-based assessments to improve fitting accuracy to a practically relevant level. Previous studies have relied on inadequate measures of agreement, such as correlation coefficients or relative reliability indices, without fully accounting for random measurement error, to justify the use of velocity profiles for 1RM estimation and individualized load prescription during training.

The results of the present study demonstrate that linear regression models systematically underestimate 1RM in youth elite athletes, whereas neural network–based approaches substantially reduced both systematic and random errors. Importantly, models incorporating additional mechanical and anthropometric information, such as the mass-velocity quotient, body mass, and body mass index, achieved the highest fitting accuracy, reflecting individual differences in leverages, neuromuscular coordination, and movement technique.

Consideration of individual performance profiles is crucial to avoid injury from overloading, which may result from premature or inaccurate predictions. Therefore, careful validation of prediction models, along with adaptation to the mechanical and anthropometric characteristics of each athlete, is essential for safe and effective implementation in strength diagnostics and training load prescription. Future research should extend these findings by testing neural network models in larger and more diverse youth populations and under truly unknown conditions to confirm robustness and practical applicability.

## Data Availability

The raw data supporting the conclusions of this article will be made available by the authors, without undue reservation.
